# “More Than Just a Manicure” Qualitative Experiences of Maternal Self-Care During COVID-19

**DOI:** 10.1089/whr.2023.0081

**Published:** 2023-12-04

**Authors:** Elizabeth S. Lax, Marianna Graziosi, Ayla N. Gioia, Vinushini Arunagiri, Sarah A. Novak

**Affiliations:** ^1^ Psychology Department, Hofstra University, Hempstead, New York, USA.; ^2^ Psychology Department, New York University, Paris, France.

**Keywords:** self-care, mothers, qualitative, social support

## Abstract

**Background:**

The COVID-19 pandemic presented families with novel challenges. Mothers were at particular risk for parental burnout, however, there is limited research on self-care behaviors to ameliorate it explicitly for mothers of young children (aged 3 and younger). Moreover, there has been little in-depth analysis on barriers to self-care and how mothers realistically implement it in their lives.

**Methods:**

In this article, we explore influences on and barriers to self-care in mothers of young children during the COVID-19 pandemic. Using a content analysis approach, we used verbal testimony from mothers to create a coding framework and applied that framework to the sample (*N* = 717).

**Results:**

Qualitative analyses revealed that beauty and personal care were the most utilized self-care acts. Two major barriers were lack of childcare and limited time. Social support was the most frequently reported asset to engaging in self-care. Participants noted that the pandemic significantly restricted their access to activities and social support.

**Conclusions:**

These findings emphasize the need to encourage mothers to prioritize self-care and incorporate their support systems to facilitate engagement. These results can inform programming to increase self-care behaviors in mothers, particularly during times of high environmental stressors. Future research should examine how resources can be allocated toward barriers of self-care to reduce burnout and improve quality of life.

## Introduction

Between 4% and 20% of mothers endorse postpartum depression in the first year after birth.^1–4^ Postpartum maternal mental health issues have far-reaching effects on fetal, infant, child, and adolescent psychological and developmental outcomes. For infants, postpartum depression can increase the risk for early challenges with emotion regulation and social behavior.^5,6^ In longitudinal studies looking at peripartum depression, an increased likelihood of child emotional problems,^7–9^ risk for clinical depression,^10^ or externalizing behavior in adolescence^11^ has been found. Moreover, although, research on mental health challenges 1 year postpartum is well-developed, it is likely that vulnerability persists beyond these early phases.

Research has demonstrated that people can remain vulnerable to depression and anxiety 3 to 5 years after giving birth^12,13^ and mothers at risk for adversity (such as experiencing social and economic challenges such as unemployment, financial problems, or parenting alone) may endorse high levels of mental health challenges even 4 or 5 years later.^14^ Furthermore, much is known about clinical levels of psychological distress 1 year postpartum, yet less attention has been given to individuals experiencing subclinical mental health challenges during this time.

The coronavirus pandemic added additional challenges for parents,^15–17^ and these disproportionally burdened mothers.^18,19^ The increase in parental mental health challenges during COVID-19 put parents, but more so mothers, at a particular risk for sacrificing their own needs to fulfill their caregiving demands. Moreover, a lack of self-care may be a significant contributor to their parental burnout.^20,21^ As stated by Dugan and Barnes-Farrell^21^ and influenced by McGowan^22^:

Self-care behaviors are decisions and actions that enhance health, prevent disease, limit illness, and restore health. They serve as a complement to formal medical care and allow people to be active agents in facilitating their health and shaping the circumstances that influence their health (p. 65).

Given the limited time and resources for mothers of young children, self-care could be a portable, widely available intervention.

During the COVID-19 pandemic, self-care and mind–body interventions garnered increased attention for this population^23^ as effective ways to bolster peri- and postpartum physical and mental health. For pregnant and postpartum individuals, these interventions are easily implementable in an at-home or self-isolation context.^24^ Facilitating engagement in self-care (protective or restorative behaviors) is a potential target to reduce vulnerability to emotional overwhelm. Recommendations to engage in self-care have become prevalent within the Western culture at large, and an emerging body of work highlights the value of these behaviors for health and well-being.^25^ However, research has also suggested that mothers of infants and young children are less able to engage in these restorative behaviors.^20,26,27^

It is likely that parents are aware of the benefits of self-care for themselves and their families, as they may have been reminded to “put your own oxygen mask on first,” because taking care of themselves will help them to better take care of others, yet many barriers can make self-care less feasible among mothers.

Research conducted with working mothers, and the “second shift” of attending to the family's needs *via* housework and childcare, further highlights the tendency of mothers to overlook their own needs. Even when mothers get to experience true leisure, it tends to be interrupted by home and family obligations.^28^ Although the task of finding time to attend to one's own needs is a significant challenge, especially for working mothers, engagement in self-care behaviors is associated with benefits not only for several well-being outcomes such as stress reduction and health promotion, but also for work-related outcomes.^21^

Yet, a limitation of this work tends to be the emphasis on physical (*e.g.*, grooming and exercise) and not on emotional self-care behaviors (*e.g.*, meditation, utilizing social support). A lack of attention toward the emotional area of well-being is often echoed in the scientific literature as well as the doctor's office for peri- and postpartum individuals. Maternal emotional self-care tends to be overlooked in general because of the emphasis placed on physical health versus mental health needs.^26^

Self-care is often mentioned as a strategy in the treatment of maternal burnout^29^ and postpartum mood and anxiety disorders,^30^ but there is a lack of systematized work about how to conceptualize it,^31,32^ predict it,^33–35^ and utilize it.^25^ A review of parental self-care studies found that across the qualitative studies, most women perceived self-care or protective health promotion behaviors as crucial to their well-being, adjustment to the maternal role, identity, and functioning.^36^ Furthermore, there is a need for this work to extend to the specific self-care needs^37^ of mothers of young children.^38^ Because this population remains vulnerable to emotional problems such as anxiety and depression 3 to 5 years postpartum^12,13^ and has limited time,^21^ a better understanding of what a tailored self-care intervention could look like is warranted.

Moreover, given that the pandemic posed challenges to parents, self-care may be one protective/restorative resource that could reduce strain in this group. However, there is little research on the topic of self-care explicitly for this population. Identifying interpersonal and intrapersonal factors that help facilitate self-care behaviors can help inform treatments for mothers facing mental health challenges during stressful life events, including the pandemic.

This study first aimed to clarify what self-care looks like explicitly for mothers of young children by using qualitative methods to build an understanding of what behaviors they engage in as self-care. In addition, this study seeks to better understand factors that help to enable mothers of young children to engage in self-care, as well as barriers that make engaging in self-care behaviors more challenging. Using qualitative analysis, we examine verbal accounts of self-care behaviors early in the COVID-19 pandemic. The results can be used to inform our understanding of facilitators and barriers to self-care behaviors in mothers of young children, particularly during times of high environmental stressors.

## Methods

### Participants

This study focuses on the content analysis of responses to open-ended questions. Participants (*N* = 808) were recruited *via* social media advertisements on Facebook and Instagram that targeted mothers in the United States. To be included, participants needed to be at least 18 years old and have a child 3 years old or younger. Participant demographic information is available in Table 1.

**Table 1.** Demographic information of the selective coding participants

Demographics	Values
Age*, M* (SD)	32.3 (5.5)
Gender, %	
Female	98.9
Nonbinary	0.6
Prefer to self-describe	0.3
Sexual orientation, %	
Heterosexual	82.5
Bisexual	11.2
Prefer to self-describe	1.7
Homosexual	1.3
Prefer not to say	0.7
Race and ethnicity, %	
White (non-Hispanic)	76.4
Black or African American	9.0
Hispanic or Latinx	8.1
Asian or Asian American	3.2
American Indian or Alaskan Native	1.8
Other	1.0
Marital status, %	
Married and cohabitating	93.1
Never married and never cohabitated	3.2
Divorced	1.7
Married, not cohabitating	0.8
Separated	0.8
Partner status, %	
Partnered	94.0
Not partner	5.7
Children, %	
One child	52.7
Two children	34.3
Three or more children	13.9

Two participants from the selective coding phase did not complete the demographic questions. *N* = 715.

### Procedure

Data were collected as part of a larger study^39^ during the early phase of the pandemic, April 25, 2020, to May 15, 2020, when ∼94% of individuals in the United States were either mandated or urged to stay-at-home to slow the spread of COVID-19.^40^ The preregistration for this project is available at https://osf.io/98xqz/ and the study was approved by Hofstra's Institutional Review Board (Register No. 20200420-PSY-HCL-WEI-1). Beyond the demographic questions, the data utilized for this study were exclusively qualitative in the form of written responses to open-ended questions about motherhood and self-care. Responses to the following questions were analyzed:

1. “Tell us about your most recent act of self-care”
2. “What barriers get in the way of your self-care? And/or what helps you to successfully engage in self-care?”
3. “Finally, we have asked several questions about how you take care of yourself. Now we are curious whether anyone (such as a friend, family member, colleague, stranger) has tried to influence the way you care for yourself since you became a parent. If this happens, please briefly describe what happens.”

The responses to each of these questions ranged from a few paragraphs to a few sentences. Given the semistructured nature of the questions, these responses offered depth of insight into the specific questions about influences on self-care, which was the aim of this article.

#### Qualitative methodology

A “directed” *content-analysis* approach^41^ was selected because the primary aim of this study was to determine the degree to which the brief written excerpts reflected existing theory on influences on maternal self-care. Another aim of the study was to use these excerpts to add to the existing theory on maternal self-care.^41^ There were three phases to the coding: (1) developing the coding framework with a subset of the data, (2) finalizing the coding framework, and (3) applying the framework to the rest of the data set. Each iteration of this coding frame and the data file are available on Open Science Framework (https://osf.io/98xqz/.).

##### Developing the framework (phase 1)

A “top-down” inductive approach,^42^ leaning on past research on maternal self-care, was used to develop the coding framework for this study. An initial framework was developed based on the relevant literature from the field, including work described above. During this first phase of coding, 91 participant excerpts were randomly selected to be independently analyzed in rounds using *Dedoose* mixed-methods software. Coders discussed how to adjust the preliminary frame to account for themes emerging from initial analysis of the data.

While the research-based initial framework provided ample scaffolding, the framework changed substantially throughout this phase. Coding continued until inductive thematic saturation^43^ was reached (*i.e.*, the framework seemed to adequately capture experiences of participants and no new codes were needed). Theme saturation was determined to be reached through discussion among coders as they iteratively adjusted the framework with each exposure to excerpts in phase 1. That is, when coders agreed that no further adjustments to the frame were needed, the framework was finalized in phase 2.

##### Finalizing the framework (phase 2)

A final coding framework (Appendix Table A1) was established, for use in the selective coding phase (described below). The finalized framework was then used in phase 3 and applied to the remainder of participants (*N* = 717). Recall, a subset of excerpts from 91 participants was used in phase 1, and therefore, they were not also included in phase 3 given that the coding team had already iteratively discussed these entries and including them could bias inter-rater reliability. Therefore, once the 91 participants from phase 1 were removed from the total sample (*N* = 808), 717 participants remained. It is the content from excerpts from this sample of 717 participants from which the results of the study are drawn.

##### Applying the finalized framework (phase 3)

In this phase, coders applied the framework to the remaining excerpts independently, without discussing the content of excerpts. In addition, coders were blind to the code application of the other members of the coding team. Throughout phase 3, each coder also noted excerpts that appeared representative of a given coding category.

Inter-rater reliability was assessed by calculating Cohen's kappa coefficient (κ) for each code (Table 2). The coding rotations were designed so that there would always be two raters applying codes to each excerpt. Overall, there was strong reliability among coders as Cohen kappa (κ) values ranged from moderate to almost perfect using the criteria set forth by Landis and Koch.^44^

**Table 2.** Code frequencies, inter-rater reliability, and strength of agreement reported by code for the complete coding framework

Categories and individual codes	Frequency of excerpts	Cohen's κ coefficient	Standard error of κ	Strength of agreement^a^
Self-care behaviors				
Beauty and personal care	274	0.899^*^	0.019	Almost perfect
Engaging in pleasurable activities	179	0.800^*^	0.026	Substantial
Personal hygiene	173	0.886^*^	0.023	Almost perfect
Being alone/solitude	135	0.752^*^	0.029	Substantial
Sleep (as self-care)	99	0.859^*^	0.031	Almost perfect
Shopping	83	0.597^*^	0.063	Moderate
Delegating childcare	63	0.670^*^	0.036	Substantial
Jogging	61	0.811^*^	0.086	Almost perfect
Exercising	55	0.883^*^	0.039	Almost perfect
Socializing	49	0.757^*^	0.045	Substantial
Making time for oneself	43	0.602^*^	0.036	Moderate
Spending time in nature	41	0.723^*^	0.050	Substantial
Taking care of health needs	41	0.752^*^	0.049	Substantial
Meditating	31	0.910^*^	0.047	Almost perfect
Yoga	19	0.949^*^	0.048	Almost perfect
Religious and spiritual activity	18	0.899^*^	0.064	Almost perfect
Reading	18	0.975^*^	0.017	Almost perfect
Delegating home and family responsibility	17	0.602^*^	0.055	Moderate
Housework/catching up on home responsibilities	16	0.724^*^	0.076	Substantial
Walking	13	0.866^*^	0.038	Almost perfect
Unwinding with a drink	13	0.832^*^	0.097	Almost perfect
Listening to music	10	0.832^*^	0.097	Almost perfect
Thinking positively	10	0.622^*^	0.077	Substantial
Barriers to self-care				
Childcare	845	0.838^*^	0.018	Almost perfect
Limited time	329	0.800^*^	0.021	Almost perfect
Home/family responsibility	265	0.754^*^	0.022	Substantial
Professional/occupational responsibility	224	0.860^*^	0.022	Almost perfect
Limited financial resources	86	0.909^*^	0.029	Almost perfect
Self-sacrificing mindset	83	0.661^*^	0.032	Substantial
Guilt	83	0.895^*^	0.035	Almost perfect
Limited social resources	60	0.595^*^	0.030	Moderate
Lack of motivation	49	0.783^*^	0.045	Substantial
Sleep/exhaustion	48	0.655^*^	0.040	Substantial
Mental health	34	0.667^*^	0.050	Substantial
Anxiety/worry	28	0.830^*^	0.062	Almost perfect
Difficulty accepting help	27	0.669^*^	0.054	Substantial
Depression	24	0.922^*^	0.051	Almost perfect
Single motherhood	21	0.873^*^	0.063	Almost perfect
Internal facilitators of self-care				
Alone time	33	0.504^*^	0.016	Moderate
Free time	18	0.529^*^	0.029	Moderate
Optimism	8	0.569^*^	0.066	Moderate
Getting sleep	7	0.498^*^	0.001	Moderate
External facilitators of self-care				
Partner	432	0.873^*^	0.018	Almost perfect
Encouragement to engage in self-care	330	0.575^*^	0.018	Moderate
Offers to delegate infant/childcare	189	0.660^*^	0.024	Substantial
Parents	168	0.873^*^	0.025	Almost perfect
Friends	104	0.812^*^	0.033	Almost perfect
Societal pressure (“they”)	78	0.641^*^	0.031	Substantial
Extended family members	65	0.767^*^	0.042	Substantial
Other moms (mom friend)	52	0.862^*^	0.042	Almost perfect
Parents in law	44	0.779^*^	0.048	Substantial
Family and friends	40	0.704^*^	0.049	Substantial
Role of COVID-19				
Influence of COVID-19	98	0.688^*^	0.031	Substantial
COVID-19 exposure	3	0.499^*^	0.001	Moderate
Quarantine	72	0.721^*^	0.037	Substantial

^a^ The strength of agreement is based on the criteria set forth by Landis and Koch^44^ where: κ < 0.00 indicates Poor Agreement, κ = 0.00–0.20 indicates Slight Agreement, κ = 0.21–0.40 indicates Fair Agreement, κ = 0.41–0.60 indicates Moderate Agreement, κ = 0.61–0.80 indicates Substantial Agreement, and κ = 0.81–1.00 indicates Almost Perfect Agreement.

^*^ An asterisk indicates that Cohen's kappa value is statistically significant at the *p* < 0.001 level.

## Results

The codes were analyzed by the frequency they were applied (Table 2). Recall, 717 participants answered 3 qualitative questions, resulting in 3 excerpts to be analyzed for each participant. Thus, each code could have been applied to a maximum of 2151 discrete excerpts. Exemplar excerpts to better illustrate the sort of responses that characterized each code are available in Appendix Table A1. The results are described first by each “branch” of the coding tree. Second, the most frequent code co-occurrences calculated by Dedoose qualitative software are discussed (Table 3) to offer insight into codes that were most likely to overlap (within and across branches).

**Table 3.** Top 15 code co-occurrences reported by frequency

Codes	Frequency
“Partner” × “Encouragement to Engage in Self-Care”	126
“Childcare” × “Home & Family Responsibility”	104
“Childcare” × “Professional/Occupational Responsibility”	90
“Childcare” × “Limited Time”	85
“Partners” × “Offers to Delegate Infant/Childcare Tasks”	75
“Home & Family Responsibility” × “Professional/Occupational Responsibility”	68
“Parents” × “Encouragement to Engage in Self-Care”	51
“Partner” × “Childcare”	48
“Limited Social Resources” × “Childcare”	43
“Parents” × “Offers to Delegate Childcare”	36
“Parents” × “Partner”	34
“Friends” × “Encouragement to Engage is Self-Care”	33
“Societal Pressure” × “Encouragement to Engage in Self-Care”	29
“Quarantine” × “Influence of COVID-19”	27

### Coding framework

There are five major branches to the framework: (1) self-care behaviors, (2) barriers to self-care, (3) internal influences that help mothers engage in self-care, (4) interpersonal influences that help mothers engage in self-care, and (5) the influence of COVID-19. Each of the branches are described.

#### Self-care behaviors

Research on self-care is limited, therefore examining what people consider self-care is important, especially when attempting to understand and support mothers with young children during times of high stress. The following codes are broken down with specific examples of what constitutes self-care for these mothers. In total, 1420 excerpts were coded for “Self-Care Behaviors,” with most excerpts coded for *Engaging in Pleasurable Activities* (*N* = 577; *i.e.*, “beauty and personal care”), followed by *Taking Care of Health Needs* (*N* = 272, *e.g.*, “taking showers is pretty much it right now” [Participant 1170]), and *Making Time for Oneself* (*N* = 268, *i.e.*, “delegating childcare”). Table 1 contains the frequency of excerpts coded for each of the remaining categories from this branch.

#### Barriers to self-care

A total of 2144 excerpts were coded for a given barrier to self-care, according to the following distribution of subcategories: *Lack of Childcare* (*N* = 845, *i.e.*, referencing time and energy spent dedicated toward caring for their children, *e.g.*, “I feel like my children get in the way of self-care such as quiet time where I could meditate or read. I cannot even shower without them banging on the door or trying to shower with me” [Participant 170]) and *Limited Time* (*N* = 818, *i.e.*, family/professional responsibilities taking time away from self-care, *e.g.*, “Too much work around the house and with the kids. Just forcing myself to just do something is hard but I do it anyway” [Participant 876]). These two barriers, *Lack of Childcare* and *Limited Time,* were the codes most frequently applied by coders to the excerpts.

Other barriers were also endorsed (*e.g.*, *Mental Health*, *Limited Financial and Social Resources*), but none of these was endorsed by more than 300 participants (for a full list of barriers see Appendix Table A1). For this branch of the coding tree, *Lack of Childcare* and *Limited Time* were the most frequently referenced barriers.

#### Personal facilitators of self-care

When it came to internal, self-directed influences to engage in self-care, a total of 66 excerpts were coded including: *Alone Time* (33), *Free Time* (*N* = 18; *e.g.*, “Hooray for naps, early bedtimes, and similar interests. Time to do what I want and sharing things that make me joyful” [Participant 570]), *Optimism* (8), and *Getting Sleep* (7).

*Alone Time* seemed to have the greatest influence on mothers' engagement in self-care as indicated by the following examples: “My husband helps me when I need time to myself” (Participant 194), “asking for help to get some alone time” (Participant 437), and “I have to schedule and plan for alone time while kids are at school and husband is working” (Participant 296). Having this alone time seemed to temporarily alleviate responsibilities: “leaving the house helps me engage in self-care so I don't jump in to help the kids or start cleaning/organizing something” (Participant 282), and “taking walks by myself and get away from people and responsibility for a little while” (Participant 286). Notably, internal influences played a much smaller role than external influences to engage in self-care (discussed next).

#### Social facilitators of self-care

A total of 1502 excerpts were coded for external influences on self-care. Participant excerpts' were most likely to refer to *Friends and Family* (*N* = 905), with *partners* (*N* = 432) being mentioned frequently. Next*, Encouragement to Engage in Self-Care* (*N* = 330) was the second-most utilized code and consisted of self-reports of friends/family members facilitating self-care. A caveat is that mothers noted that sometimes this encouragement falls short when it is unwelcomed or insincere (*e.g.*, “I have been encouraged more to take time for myself, but no one has provided solutions on how to do that or offered help” [Participant 632]). *Offers to Delegate Childcare* (*N* = 189) and *Societal Pressure* (*N* = 78) were also endorsed as influences. The numbers reflect the tremendous impact that family and friends have on mothers' self-care.

#### COVID-19

A total of 173 excerpts had mentioned COVD-19, including: *Influence of COVID-19* (*N* = 98, *i.e.*, “loss of access to childcare”), *Quarantine* (*N* = 72, *e.g.*, “The shutdown keeps me from engaging in social activities or getting a massage or having time away from my baby. There is no more work-life balance. Work and life are happening at the same time all the time” [Participant 530]), and *COVID-19 Exposure* (*N* = 3). For the purposes of coding, any excerpts with the phrase “COVID-19” or “pandemic” were coded for *Influence of COVID-19,* and any that mentioned “quarantine” or “lockdown” were coded for *Quarantine.*

### Code co-occurrence: trends

The previous section reflects the number of excerpts coded for a single theme, however, we were also able to identify codes that tended to co-occur. The top 15 co-occurrences are represented in Table 3, and the top 5 are as follows:

1. “Partner” × “Encouragement to Engage in Self-Care” (*e.g.*, “My husband gives me the time I need to work and do things for myself. This is invaluable.” Participant 95);
2. “Lack of Childcare” × “Home & Family Responsibility” (*e.g.*, “Full-time mom duty and housework makes it tough. By the time all my jobs are done and the baby's sleeping I'm too tired to do stuff I like” Participant 439);
3. “Lack of Childcare” × “Professional/Occupational Responsibility” (*e.g.*, “Working from home, both my husband and myself, and taking care of my 1 year old” Participant 238);
4. “Lack of Childcare” × “Limited Time” (*e.g.*, “Busy life with young kids makes it hard to find time” Participant 181); and,
5. “Partners” × “Offers to Delegate Infant/Child tasks” (*e.g.*, “My husband frequently tells me to go and relax and that he'll take care of our child” Participant 228).

These data lend some insight into how the codes show up in mothers' accounts of self-care. Childcare and the activities that interfere with it feature prominently among the top code co-occurrences. Furthermore, partners and their influence in encouraging self-care, as well as offering to delegate childcare, are influential in facilitating self-care. These themes extend throughout the top 15 co-occurrences (Table 3), where social support targeted at encouraging self-care and mitigating the responsibilities of childcare and home is salient.

## Discussion

This study aimed to explore the experience of mothers of young children during the COVID-19 pandemic, specifically in terms of self-care. Through qualitative analysis, we highlighted activities that were most likely to be utilized for self-care, identified barriers to self-care, as well as facilitating factors. We also describe the ways in which the pandemic influenced self-care for the mothers in our study. Perhaps most importantly, the results highlight the crucial role of a mother's support system.

### Self-care behaviors

Our participants reported engaging in pleasurable activities as a primary mode of self-care. They endorsed using beauty and personal care (*i.e.*, getting a pedicure) as well as personal hygiene (*i.e.*, shower). These modes of self-care have a restorative and somatic quality and, in theory, should not take much time. Yet, we highlight this because it seems to be the case that for mothers of young children, even the smallest acts within one's day are perceived to be acts of self-care. This is important for interventions aimed at increasing self-care, which may set unrealistic goals. Brown et al.^45^ found that while mothers may want to spend more time on leisure (*i.e.*, activities for pleasure or exercise), barriers such as limited time and energy, as well as a self-sacrificing mindset, get in the way. Indeed, addressing these barriers is important, however, building up the “small wins” may be of equal or greater value.

There is limited research on the specific self-care behaviors of mothers of young children, however, our findings offer a glimpse into what is utilized (as opposed to what is prescribed or even desired). As one mother described: “I consider washing my face in the AM and PM self-care” (Participant 891).

### Barriers to self-care

Extant literature has shown that limited time, lack of childcare, and limited resources are common barriers to self-care for mothers.^21,46^ Our study further advances these findings by examining these barriers within the context of the COVID-19 pandemic. This could be attributed to the amount of time women are expected to spend on home and family work. Some studies indicate that women spend about twice the time on this work as men.^47,48^ Mothers have also been found to spend more time on childcare than fathers.^49–51^ Moreover, Dugan and Barnes-Farrell^22^ reported that these barriers might be more salient for working mothers who have a *second shift*, where mothers share the primary responsibility for their children and home in addition to their *first shift* of paid work responsibilities.

These additional burdens might explain why time and limited resources related to childcare are still the biggest barriers for mothers to engage in self-care. This again illustrates how for this population self-care must happen quickly and “between the cracks” of packed daily routines.

### Social support

Overwhelmingly, what enabled the mothers in our study to move beyond quick and spontaneous self-care behaviors was social support. This aligns with much of the existing literature on maternal self-care, which has indicated that social support is a crucial component to encouraging mothers to engage in leisure activities^45^ and to protect against the influence of chronic stressors.^52–54^ In our study, family and friends' influence on self-care was mentioned more than any other item in the entire coding tree. By far, partners played the biggest role in influencing self-care for the mothers in our study and encouragement was often reported. This fits with previous research that has also conveyed that asking partners to care for the infant or encouragement from partners to engage in self-care is an important facilitator of maternal self-care.^46^

Future interventions should consider including partners and parents as part of their models since they play a crucial role in facilitating self-care. More importantly, however, the U.S. culture frequently discusses self-care and seems to value it, yet there is rarely mention of the barriers or social resources that make self-care possible, especially among mothers of young children. These findings give concrete direct examples of why it can be difficult to achieve and where we can intervene.

### COVID-19

A total of 173 of the excerpts had a mention of COVD-19, including: *Influence of COVID-19* (98), *Quarantine* (72), and *COVID-19 Exposure* (3). For the purposes of coding, any excerpts with the phrase “COVID-19” or “pandemic” were coded for *Influence of COVID-19,* and any that mentioned “quarantine” or “lockdown” were coded for *Quarantine.* The themes seem to be similar regardless of which language was used, and therefore, these codes are discussed together below.

The COVID-19 pandemic was associated with an increase in both professional and social life stressors for parents^17^ and increased vulnerability to stress and mental health difficulties.^17,18^ This was clearly present in our sample (see the *Mental Health* category of Appendix Table A1). In fact, while limited time seems to have been a barrier for mothers before the pandemic, the idiosyncrasies of government-imposed quarantines and social distancing further blurred the lines between work, life, *and* parenting for the mothers in our sample.^55,56^ One mother describes the layers of challenge, “Caring for a toddler at home while daycare is closed and also needing to work from home, while maintaining the household” (Participant 184).

The pandemic not only amplified the limited time barrier but also restricted access to social support and childcare resources (daycare, babysitters, etc.^57,58^), which are crucial influences on self-care in mothers of young children. As Participant 371 describes, the pandemic put parents in the position of having to choose safety over social support: “Having a newborn baby makes it hard to make time for myself. Also because of the pandemic we are choosing to self-isolate and not seeing family or friends in person.”

Finally, parents reported feeling increased pressure to implement academic programming while children were learning remotely during the pandemic.^59^ Certainly not every environmental stressor might be as functionally impairing as the COVID-19 pandemic, but future interventions should consider how these environmental stressors amplify existing barriers, dilute or interfere with protective influences, and how they may create new barriers. In addition, it remains unclear how these stressors experienced during the pandemic may persist or change over time. COVID-19 likely has changed the landscape for remote/hybrid models of employment,^60^ thus potentially influencing mothers' likelihood to work from home, which may have a downstream influence on self-care.

### Strengths/limitations

Our study had several strengths that yield important implications for practice and research. First, we examined barriers for self-care behaviors during a period of unique stress. Our findings suggest that COVID-19 had a direct impact on social support, which was one of the most salient self-care barriers. Social support was reported to be important in not only facilitating self-care (*e.g.*, family/friends offering childcare while mothers engage in self-care), but also was reported to be a pleasurable activity that mothers engage in for self-care (*e.g.*, spending time with friends). COVID-19 may have impacted not only the facilitation of self-care behaviors, but also prevented some self-care behaviors due to pandemic-related guidelines. Mothers therefore reported adapting their self-care behaviors to include socialization through online platforms.

Previous research has demonstrated that online socialization can facilitate a positive impact on social support and shared experiences for mothers.^61,62^ Future research could explore the efficacy of online programs that promote social connection as well as other self-care behaviors since virtually delivered interventions are likely more accessible for this population. Furthermore, this study confirmed previous findings that limited time, lack of childcare, and limited resources continue to be barriers to self-care.^21,46^ This indicates that interventions aiming to influence self-care with this population would benefit from continued focus on strategies that can mitigate these barriers such as including social supports in self-care scheduling/troubleshooting, helping mothers to reframe their self-care expectations, and teaching acceptance skills,^30,63,64^ such as mindfulness to increase self-compassion and flexibility particularly during times of high stress.

The present study also had some limitations. A limitation is that the initial, hypothesized coding framework did not capture all major trends. The trend of *encouragement to engage in self-care having a negative impact or influence* was not initially captured and requires further investigation, given the importance of this as a possible intervention point. Since we did not anticipate this in our coding, we are unable to provide an exact number of excerpts for which this negative valence appeared, however, several exemplars of what “falling short” is similar to, as reported by mothers in their excerpts, are provided here. For some mothers, this sort of encouragement is unwelcome, as illustrated here, “My husband tells me to do something for myself and I tell him to not tell me what to do with my life” (Participant 448).

Many mothers also noted that encouragement often seemed superficial as they cited a need for more support, resources, or action: “I keep being told to relax and take it easy, but my house is a mess, and my baby needs to be taken care of. I would relax if other people would take initiative to do things to help me instead of waiting for me to tell them every time. I am so tired” (Participant 817). “My aunt insists I take the time to take an Epsom salt bath/shave/cook for myself but fails to help with the baby in any meaningful capacity so that I can do so. Her semi-constant reminders that I'm not able to engage in the kind of self-care regimen I kept pre-baby without offering any meaningful support so that I can do so only serve to exacerbate my frustrations, sense of isolation, extreme responsibility to my child, depression, and worthlessness” (Participant 527).

These negatively received words of encouragement speak to the importance of how social support is conceptualized and experienced.^65^ As demonstrated above, encouragement to engage in self-care, when it falls short, may perpetuate negative affect and views of the self. This is in line with prior work on miscarried support that highlights that well-intentioned communication can have negative effects on an individual's emotions and furthermore create distance in relationships.^66^

Also, by looking closely at the discrete experiences of mothers of young children, our coding framework does not account for the systemic and social forces that may uphold barriers to self-care.^26^ Our sample was not racially diverse, and we did not consider experiences that are affected by race, socioeconomic status, employment status, or single motherhood. By focusing on the majority's experience, our study may have missed the minority experiences, which are equally as important. This is a crucial point and one that merits serious consideration by researchers and clinicians.

Given the uniqueness of the pandemic, findings cannot generalize readily to other times in history, however, they may offer insights into post-COVID challenges (*e.g.*, remote work and schooling; hybrid schedules) that lie ahead for this population. While this may help us to understand how mothers respond to environmental stressors, it is nevertheless possible that mothers may respond differently to the unique aspects of the early months of the COVID pandemic (such as quarantine) than more mild stressors. Future researchers might explore the degree to which these protective influences and barriers operate in the lives of mothers of young children in other kinds of environments with varying degrees of stress.

## Abbreviations Used

AM: morning, or ante meridiem
PM: evening, or post meridiem

## Appendix

**Appendix Table A1.** Coding framework and exemplar excerpts for each code

Self-care behaviors	
Making time for oneself	
Delegating childcare	“Asking my husband to get up with the baby so I could get needed sleep” (632)
Delegating home/family responsibility	“Yes, my husband always encourages and supports me to get care of myself. He helps me to do chores so I can get sometime for myself.” (140)
Being alone/solitude	“Saying no to a social zoom with family so I could have quiet time by myself instead” (1182)
Thinking positively	“I have a monthly call with friends to discuss personal goals and accomplishments” (202)
Taking care of health needs	
Personal hygiene	“Taking showers is pretty much it right now” (1170)
Sleep	“Taking a one-hour nap during the day” (530)
Engaging in pleasurable activities	
Beauty and personal care	“Pedicure while visiting my parents and they watched my daughter” (864)
Unwinding with a drink	“Drank and gossiped via video chat with a friend” (875)
Listening to music	“Listening to music while I do the dishes and leave the baby with my husband” (105)
Reading	“I re-read my favorite book.” (1180)
Shopping	“I bought new clothes” (633)
Exercising	
Meditating	“I have a counselor who is teaching me mindfulness and staying in the present. My husband highly encourages my self-care.” (402)
Walking	“Going for a walk with my family.” (635)
Jogging	“Went for a run today.” (734)
Yoga	“My husband started giving our newborn a bottle every afternoon so I can go have 1–2 hours of free time. Yesterday I took a short walk in the woods and did yoga” (492)
Housework/catching up on home responsibilities	“I let my husband take care of the kids so I could go to the grocery store.” (656)
Religious and spiritual activity	“Reading scriptures in the morning” (179)
Spending time in nature	“Played yard games outside in the sunshine” (1483)
Socializing	“I did a video call with my brothers” (534)

Barriers to self-care	
Limited time	
Home/family responsibility	“Too much work around the house and with the kids. Just forcing myself to just do something is hard but I do it anyway.” (876)
Professional and occupational responsibility	“No time between a demanding job and a young toddler!” (530)
Limited financial resources	“People say go out more for one away time but it's hard when no one can watch the baby and you don't have money for a baby-sitter” (686)
Limited social resources	“I have no one to help with my daughter.” (864)
Single motherhood	“I'm a single mom of a 10-month-old; I have zero help and zero personal time/space. Sesame Street occasionally distracts the baby long enough to let me eat, breathe fresh air, or go to the bathroom.” (527)
Difficulty accepting help	“Feeling guilty for asking my mother to take care of my child for me too. Husband doesn't help me as much as I wished he did.” (637)
Lack of childcare	“I feel like my children get in the way of self-care such as quiet time where I could meditate or read. I cannot even shower without them banging on the door or trying to shower with me.” (170)
Mental health	
Anxiety/worry	“My daughter has severe separation anxiety, and seeing her anxious makes me feel anxious even when I know she is being well taken care of.” (416)
Depression	“Depression gets in the way. Therapy helps, along with my support system.” (635)
Guilt	“The guilt is what I struggle with the most, so when someone else suggests I do something, or they plan something it's easier to do it.” (311)
Lack of motivation	“Just lack of motivation. when I get to it, I'll get to it” (472)
Self-sacrificing mindset	“The baby's needs are above my own right now. He's never out of my thoughts.” (106)
Sleep/exhaustion/lack of energy	“Lack of energy after caring for my children, my home, my work.” (641)

What helps mothers engage in self-care (personal)	
Optimism	“Keep my mind positive to live positively.” (140)
Free time	“Hooray for naps, early bedtimes, and similar interests. Time to do what I want and sharing things that make me joyful.” (570)
Alone time	“I need more time alone and out of the house” (845)
Getting sleep	“Husband, he'll take the baby and let me get some sleep” (240)

What helps mothers engage in self-care (social)	
Family and friends	
Parents	“My parents, and in laws sometimes take my son off our hands so my husband and I can have some time alone to appreciate ourselves and one another. We sometimes spend time doing whatever we want to do, whether individually or together.” (182)
Parents-in-law	“My mother-in-law has been very good about helping to alleviate some of the things on my “to do” list. For example, when she babysits our kids she will often also do dishes, do laundry, cook dinner, whatever needs to be done really. It always makes me feel much more relaxed to come home and see those things that needed to be done, done.” (142)
Extended family members	“Two children and husband. My parents and some extended family help me out when I need it.” (656)
Partner	“My husband tries to encourage me to eat and relax. He is always willing to take over our daughter's needs so I can have some time to myself.” (399)
Friends	“A close friend encourages me to seek therapy to work on my personal things and another to take time out for me.” (442)
Other moms (mom friend)	“Other moms, encouraging the small things like a shower or painted nails to help myself feel more human and happy.” (176)
Offers to delegate infant/childcare	“My mom tries to help me by offering to watch my baby so I can take naps, or work out.” (235)
Encouragement to engage in self-care	““Every now and then my husband thinks to say “hey you should take this money and go treat yourself” to either clothes, shores, massage, hair, nails, whatever I decide I want to use it for” (511)
Societal pressures (“They”)	“Modern culture influences the way I view taking time for myself. I feel women are made to feel guilty for taking time to themselves and for falling short of “doing it all.” This influences my decisions on how much time I take for myself and how guilty I feel about taking time away from my family.” (611)

Influence of COVID-19	
Influence of COVID-19	“Yes, many people have told me I need to make time for myself but it is extremely hard to do so, and next to impossible to do so during the COVID-19 pandemic.” (530)
COVID-19 exposure	“Having an at-risk person in my household” (204)
Quarantine	“The shutdown keeps me from engaging in social activities or getting a massage or having time away from my baby. There is no more work-life balance. Work and life are happening at the same time all the time.” (621)
